# *Leptosphaeria maculans* Alters Glucosinolate Accumulation and Expression of Aliphatic and Indolic Glucosinolate Biosynthesis Genes in Blackleg Disease-Resistant and -Susceptible Cabbage Lines at the Seedling Stage

**DOI:** 10.3389/fpls.2020.01134

**Published:** 2020-07-30

**Authors:** Arif Hasan Khan Robin, Rawnak Laila, Md. Abuyusuf, Jong-In Park, Ill-Sup Nou

**Affiliations:** ^1^Department of Horticulture, Sunchon National University, Suncheon, South Korea; ^2^Department of Genetics and Plant Breeding, Bangladesh Agricultural University, Mymensingh, Bangladesh; ^3^Department of Agronomy, Patuakhali Science and Technology University, Patuakhali, Bangladesh

**Keywords:** blackleg disease, *Leptosphaeria maculans*, glucosinolates, seedling resistance, cabbage, expression analysis

## Abstract

The fungal pathogen, *Leptosphaeria maculans* causes a severe and economically important disease to *Brassica* crops globally, well-known as blackleg. Besides, the anti-oxidative defense response of glucosinolates to fungal pathogens is widely established. Despite notable importance of glucosinolates in blackleg disease resistance the association of glucosinolate pathway genes in glucosinolate mediated defense response after *L. maculans* infection remains incompletely understood. The current study was designed to identify glucosinolate-biosynthesis specific genes among the eight selected candidates induced by *L. maculans* and associated alterations in glucosinolate profiles to explore their roles in blackleg resistance at the seedling stage of cabbage plants. The defense responses of four cabbage inbred lines, two resistant and two susceptible, were investigated using two *L. maculans* isolates, 03-02s and 00-100s. Pathogen-induced glucosinolate accumulation dynamically changed from two days after inoculation to four days after inoculation. In general, glucosinolate biosynthetic genes were induced at 24 h after inoculation and glucosinolate accumulation enhanced at two days after inoculation. An increase in either aliphatic (GIB, GRA) or indolic (GBS and MGBS) glucosinolates was associated with seedling resistance of cabbage. Pearson correlation showed the enhanced accumulation of MGBS, GBS, GIB, GIV and GRA after the inoculation of fungal isolates was associated with expression of specific genes. Principal component analysis separated two resistant cabbage lines—BN4098 and BN4303 from two susceptible cabbage lines—BN4059 and BN4072 for variable coefficients of disease scores, glucosinolate accumulation and expression levels of genes. Enhanced MGBS content against both fungal isolates, contributing to seedling resistance in two interactions—BN4098 × 03-02s and BN4303 × 00-100s and enhanced GBS content only in BN4098 × 03-02s interaction. Aliphatic GRA took part in resistance of BN4098 × 00-100s interaction whereas aliphatic GIB took part is resistance of BN4098 × 03-02s interaction. Aliphatic GIV accumulated upon BN4098 × 03-02s interaction but *GSL-OH*-*Bol033373* and *CYP81F2*-*Bol026044* showed enhanced expression in BN4303 × 03-02s interaction. The association between the selected candidate genes, corresponding glucosinolates, and seedling resistance broaden the horizon of glucosinolate conciliated defense against *L. maculans* in cabbage seedlings.

## Introduction

Blackleg is a devastating disease of *Brassica* crops that causes nearly one billion dollar crop losses every year, globally (Howlett, 2004; Fitt et al., 2006; Fitt et al., 2008). *Leptosphaeria maculans*, a hemi-biotrophic fungal pathogen, causes blackleg disease. Upon severe infestation of this pathogen, often complete loss of oilseed canola and rapeseed (*Brassica napus*) is reported (Li et al., 2003; Rouxel et al., 2003; Sprague et al., 2006). Blackleg disease is equally devastating to several subspecies of *B. oleracea*, including cabbage (Humpherson-Jones, 1985; Rico et al., 2001; Dilmaghani et al., 2010; Dilmaghani et al., 2013; Piliponyte-Dzikiene et al., 2015). Notably, this disease became epidemic in cabbage in Wisconsin, USA about a century ago (Henderson, 1918). Developing sustainable genetic resistance is the most suitable way of protecting *Brassica* germplasm from the devastation of blackleg disease despite the availability of chemical control methods (Del Rio and Ruud, 2013; Fraser et al., 2016; Koh et al., 2016; Potter et al., 2016).

In plants, a few secondary metabolites offer general resistance to insects and pathogens other than *R*-gene mediated race-specific resistance (Wink, 1988; Giamoustaris and Mithen, 1997; Tierens et al., 2001; Brader et al., 2006; Lattanzio et al., 2006; Evivie et al., 2019). Glucosinolates (GSLs) are the renowned secondary metabolites produced in *Brassica* crop plants which have pivotal importance in resistance of plants against pathogens and insects through their anti-oxidative properties (Hogge et al., 1988; Mithen et al., 1995; Benderoth et al., 2006; Hopkins et al., 2009). The Brassicaceae family members produce aliphatic, inodolic and aromatic glucosinolates (Fahey et al., 2001; Mithen, 2001; Bekaert et al., 2012). Breakdown products of these sulfur- and nitrogen-containing glucosinolates are primarily isothiocyanates and sulforaphane. These compounds upon the hydrolysis of endogenous myrosinase enzymes (β-thioglucoside glucohydrolases) become anti-oxidative and anti-fungal in plants (Chew, 1988; Giamoustaris and Mithen, 1995; Manici et al., 1997; Agerbirk et al., 1998; Brader et al., 2001; Tierens et al., 2001; Barth and Jander, 2006; Stotz et al., 2011; Calmes et al., 2015).

Despite general importance of glucosinolates against fungal pathogen, specific role and association between glucosinolate contents and resistance of the *B. oleracea* subspecies to various fungal pathogens remain obscure. A few studies, in nineties, reported no strong correlation between glucosinolates in different *Brassica* species and resistance to *L. maculans* (Mithen and Magrath, 1992; Sexton et al., 1999). Moreover, a negative association between glucosinolate contents and *Alternaria* infection in *B. napus* was reported (Doughty et al., 1991; Giamoustaris and Mithen, 1997). By contrast, Li et al. (1999a) in *B. napus*, reported a positive correlation between indolic glucosinolates induced upon the infection of *Sclerotinia sclerotiorum* and resistance of plants. In cabbage, inoculation of both resistant and susceptible plants with *S. sclerotiorum* and *Mycosphaerella brassicicola* revealed that both aliphatic and indolic glucosinolates raise in contents in the resistant lines (Abuyusuf et al., 2018a; Abuyusuf et al., 2018b). Furthermore, a recent study described possible mechanisms of overcoming glucosinolate–myrosinase–isothiocyanate defense system of *Phoma lingam* and *Verticillium dahliae* as these fungi species may degrade some primary glucosinolates to a less toxic level (Rahimi and Siamak, 2020). The apparent contradictions in literature may reflect the fungal lifestyles—e.g., necrotrophes and biotrophes (Sanchez-Vallet et al., 2010), type of hosts (Buxdorf et al., 2013), genetic identity of hosts—e.g., homozygous and heterozygous plant populations. In addition, against the *L. maculans*, majority of the earlier studies explored a relationship between total glucosinolates, rather than individual glucosinolate components, and resistance of plants (Mithen and Magrath, 1992; Giamoustaris and Mithen, 1997; Sexton et al., 1999; Kliebenstein et al., 2002).

By contrast, comparatively recent studies showed that upon infection of the hemibiotrophic fungus *L. maculans* in *B. rapa* both aliphatic and indolic glucosinolate compounds were induced (Abdel-Farid et al., 2010; Robin et al., 2017a). Similarly, in Brassicaceae family members, induced indolic glucosinolates exhibited association with resistance to both biotrophs and necrotrophs besides hemibiotrophs (Bednarek et al., 2009; Hiruma et al., 2013). Further, induced contents of 4-methoxy-glucobrassicin (MGBS) upon infection of fungal pathogens indicated that glucosinolate–myrosinase system has vital role in plant’s defense. In Arabidopsis, the expression of *CYP81F2* gene, activated by myrosinase PEN2, was found to regulate the accumulation of 4-methoxy-glucobrassicin (Bednarek et al., 2009).

In our previous study with adult cabbage plants, *L. maculans* infection induced glucosinolate-biosynthesis genes in cabbage, with concomitant changes in individual glucosinolate contents. In resistant lines, both aliphatic and indolic glucosinolates are associated with resistance, with aliphatic glucoiberverin (GIV) and glucoerucin (GER) and indolic glucobrassicin (GBS) and MGBS glucosinolates particularly important (Robin et al., 2017a). This study also reported that resistance response of cabbage inbred lines differs between seedling stage and adult stage. Despite general belief that accumulation of glucosinolate may vary dynamically at different time intervals due to plant–microbe interactions, majority of the previous studies measured glucosinolate accumulation and the expression of glucosinolate biosynthesis genes at single time-point after inoculation of *L. maculans* at the seedling stage. The present study was, therefore, explored the expression profile of glucosinolate biosynthesis genes at three different time-points and measured glucosinolate accumulation at two different time-points after the inoculation of two isolates of *L. maculans* at the seedling stage. This study also explored a tri-angular association among expression levels of glucosinolate biosynthesis genes, accumulation of glucosinolate compounds and resistance response of plants to draw a conclusion. The results of the present study shed-light the existing contradictions in role of glucosinolates in glucosinolate-myrosinase system during *L. maculans*–host plants interaction.

## Materials and Methods

### Plant Materials and Growth Conditions

Seeds of four cabbage inbred lines were sown in 32-celled trays in a plant culture room. Two cabbage lines—BN4098 and BN4303 were reported to be resistant and two other lines—BN4059 and BN4072 were susceptible at the seedling stage (Robin et al., 2017b). The soil mixture used for sowing seeds was composed of peat moss, coco peat, perlite, zeolite, and vermiculite. Seedlings were allowed to grow at 20 ± 2°C temperature, 16:8 h day: night, 65% relative humidity and at a light intensity of ca. 400 μmol m^−2^ s^−1^ (Yi et al., 2015). Florescent light bulbs were used as the light source.

### *L. maculans* Isolates and Seedling Inoculation

Hypersensitivity response of two fungal isolates used to inoculate cabbage inbred lines including culturing technique and methods of spore preparation have been published previously (Robin et al., 2017b). Two isolates of *L. maculans*, 03-02s (*AvrLm1-4-6-7-11-J1-S*, *AvrLep1-2-3*) and 00-100s (*AvrLm2-3-6-9-J1-S*, *AvrLep1-2*), were collected from Agriculture and Agri-Foods (AAFC), Saskatoon, Canada. Fungal isolates were cultured in 20% V_8_ medium for 10 days for spore preparation. After 10 days, spore suspension was prepared by overflowing 10 ml of sterile distilled water to each plate and scraping with a sterile microscope slide. To remove the mycelia and other debris, the spore suspension was filtered using sterile Miracloth (EMD Millipore Corporation, USA). The filtered spore suspension was concentrated before preparing a spore solution of 2 × 10^7^ spores ml^−1^ concentration through dilution. Seedlings were inoculated twice with the prepared spores—at 10 and 26 days age of the plants. At 10 days age of the seedlings, 10 µl spores was inoculated at the center of each cotyledon of a seedling. At 26 days age of the seedlings, approximately four wounds were created per cm^2^ leaf area in all true leaves including cotyledons and 10 µl spores was inoculated per wound. There were six replicates in each experiment for control, mock-treatment (wounded only) and inoculation with 03-02s and 00-100s isolates.

### Collection and Preparation of Leaf Samples for Analysis

Two separate sets of plants, grown simultaneously at the same plant culture room in the same growing conditions, were inoculated at 26 days old plants—one set is to collect samples to estimate relative expression levels of glucosinolate biosynthesis genes and another set is to measure glucosinolate accumulation. Samples of three biological replicates were kept for each analysis. In this study, we infected 26 days old plants when cotyledons were still alive. We inoculated both cotyledons and true leaves at the time of inoculation. For HPLC analysis, a large amount of samples are required—about 10 g for each biological replicate (Yi et al., 2016). If we infect only cotyledons of the 7–10 days old seedlings we need hundreds of plants to obtain that amount of samples. For this reason we allowed cabbage seedlings to grow 26 days before inoculation, so that sufficient samples can be obtained from the infected plants for the HPLC analysis.

Relative expression levels of genes were measured at 6, 24 and 48 h (2 dpi, days post inoculation) after inoculation in control, mock-treated and *L. maculans* inoculated plants. By contrast, glucosinolate accumulation was measure at 2 and 4 dpi in control, mock-treated and *L. maculans* inoculated plants. The samples collected for HPLC analysis and real-time PCR were flash-frozen in liquid nitrogen and immediately stored at −80°C.

### Primer Design for Expression Analysis of Glucosinolate Biosynthesis Genes

**Figure 1:**
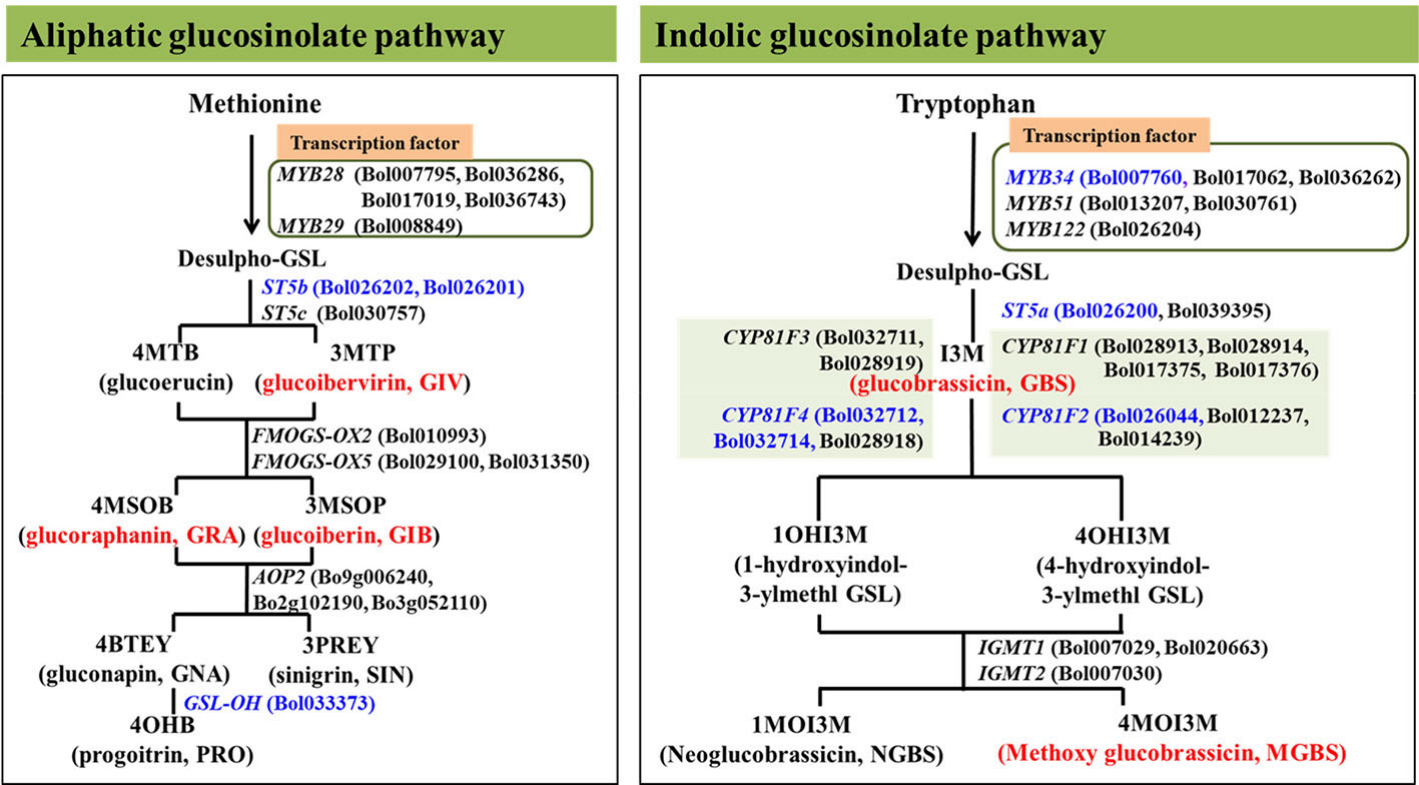
Position of eight aliphatic and inodolic glucosinolate (GSL) biosynthetic genes analyzed in this study in glucosinolate biosynthesis pathway represented by blue colors. Red color represent the glucosinolates were altered in response to enhanced expression of the analyzed genes in this study (Robin et al., 2016; Robin et al., 2017a).

Eight genes involved in glucosinolate biosynthesis were selected for transcription analysis to determine how transcript levels are affected by pathogen inoculation (**Table 1**, **Figure 1**, Robin et al., 2017a). One of the gene encode indolic transcription factors, three are aliphatic biosynthesis genes and four are indolic biosynthesis genes (Robin et al., 2016; Yi et al., 2016). The efficiency of designed primers was tested according to Robin et al. (2016).

**Table 1:** Primer sequences for glucosinolate-biosynthesis related genes used for relative expression analysis through qPCR in 26 days old plants inoculated with 03-02s and 00-100s isolates (Robin et al., 2016 and Yi et al., 2016).

Gene Name	Accession Number	cDNA Size (bp)	Forward Primer Sequence	Reverse Primer Sequence	Product Size (bp)
			**Aliphatic biosynthesis-related genes**		
*ST5b*	Bol026201	1,035	CCGAGCCGTCAGAATTCAAG	GCTATGGCGAAAGTGAGAGC	247
	Bol026202	1,035	AAGCCTTGACTTTCGCCATC	ACTTCACAACTGAGTCCGGT	204
*GSL-OH*	Bol033373	243	GATTGTGCAAAAGGCTTGT	AGAGCATTAGGATTAGGAGGA	188
			**Indolic transcription factor-related gene**		
*MYB34*	Bol007760	843	TGAAGGAGGATGGCGTACTC	CAGTTCGTCCCGCCAAATTA	203
			**Indolic biosynthesis-related genes**		
*ST5a*	Bol026200	1,017	GTCCGGTTGCAAGATGGTTT	CCTCTCCGGGTTCTCTTTGT	214
*CYP81F4*	Bol032712	1,506	CGGTGGAGGAGAAGGAGAAA	CTGACACATGGCTCGTAACG	226
	Bol032714	960	ACCCTGGTGAATACTTGCCA	GAAACACACTGAAGCAGAAC	239
*CYP81F2*	Bol026044	1,482	TTCTCCCTACGTTACGGCTC	CTACGAACGGAGAGGAGTCC	251

### cDNA Synthesis and Real-Time Quantitative PCR Analysis

Total RNA of the collected samples was extracted using RNeasy mini kit, Catalog No. 74106, Qiagen, Valencia, CA, USA. PrimeScript-based kit (Takara Bio, Inc., Shiga, Japan) was used to synthesize cDNA from total RNA. iTaqTM SYBR^®^ Green Super-mix was used with ROX (Bio-Rad, Hercules, CA, USA) to conduct quantitative RT-PCR (qPCR). A total reaction volume of 20 μl was prepared with PCR master mix 10 μl, ultra-pure water 7 μl, forward and reverse primers 2 μl and cDNA template (concentration of 60 ng μl^−1^) 1 μl for each reaction. PCR conditions were as follows: denaturation 10 min at 95°C, 40 cycles of denaturation 20 s at 95°C, annealing 20 s at 58°C, and amplification and elongation for 30 s at 72°C. Data were taped as fluorescence for each sample at the end of each of 40 cycles. Each biological replicate was repeated in three technical replicates. LightCycler96 software (Roche, Mannheim, Germany) was used for quantification cycle (Cq) analysis. Livak’s comparative 2^−ΔΔ^*^Ct^* method was used to quantify the relative transcription of each sample (Livak and Schmittgen, 2001). Three different *actin* genes were expressed in all inbred lines i.e. GenBank Accession Nos. AF044573 (Zhang et al., 2012), JQ435879 (Nawaz et al., 2014), and XM_013753106 (Lee et al., 2015), were used as a reference.

### Measurements of Glucosinolate Contents

Desulfoglucosinolates were collected from three biological replicates of cotyledon samples for each of the control, mock-treated, and *L. maculans*-infected plants, *via* a modified HPLC protocol as previously described (Yi et al., 2015; Robin et al., 2016; Yi et al., 2016). Frozen samples stored at −80°C was treated with methyl alcohol and then ground to a fine powder. The powdered samples were preserved for 10 min at 70°C and then kept about 1 h at room temperature and centrifuged for 8 min at 10,000×*g* at 4°C to remove sediments (structural components and protein molecules). The supernatant fluid was carefully passed over an anion-exchange column. The action of centrifugation and anion-exchange chromatography was repeated two more times. The supernatant separated at the end of anion-exchange chromatography was treated as crude glucosinolate sample. For desulfation, this crude glucosinolates were used. In desulfation process, barium acetate (0.5 ml 50 mM) and lead acetate (0.5 ml 50 mM) were added with the crude glucosinolates and then centrifuged for 10 min at 2,000×*g*. The upper fluid was then passed through a pre-equilibrated (with 0.5 M sodium acetate) DEAE-Sephadex column. Desulfation was started by the addition of aryl sulfatase at 250 μl to the column and was permitted to run for 16 h. The desulfated glucosinolates were then eluted with the help of 1 ml distilled water. The eluted solutions were purified by centrifugation at 20,000×*g* for 4 min at 4°C and filtration by a PTFE filter (13 mm, 0.2 μm, Advantec, Pleasanton, CA, USA). The purified solutions were then allowed to HPLC analysis. Individual glucosinolate compounds were determined by a PDA 996 UV-visible detector (Waters) at of 229 nm wavelength. For quantification of the detected glucosinolates, a standard curve was used which was prepared from commercial sinigrin (SIN). Mass spectrometry analysis (HPLC/MS, Agilent 1,200 series, Agilent Technologies) was done to identify individual glucosinolate component (Yi et al., 2016; Abuyusuf et al., 2018a).

### Statistical Analysis

A two-way analysis of variance was conducted to test the statistical significance among genotypes (cabbage lines), treatments and genotypes x treatments using Minitab 18 statistical software (Minitab Inc., State College, PA, USA). A *posthoc* Tukey’s pairwise comparison was conducted to explore statistical significance among treatment, genotypes and interactions. Test statistic, degrees of freedom, and p-values of statistical significance for relative expression of biosynthesis genes and glucosinolate contents are given in **Tables S1** and **S2**, respectively.

Pearson correlation coefficient was estimated between glucosinolate content of each category—(aliphatic and indolic) and expression of biosynthesis genes under each category. Principal component analysis (PCA) was conducted taking disease scores of four genotypes, expression levels of genes and glucosinolate contents of control plus mock, 03-02s and 00-100s treatments at 2 days after inoculation (d2).

## Results

### Resistance of Selected Cabbage Lines to *L. maculans* Infection at Seedling Stage

**Figure 2:**
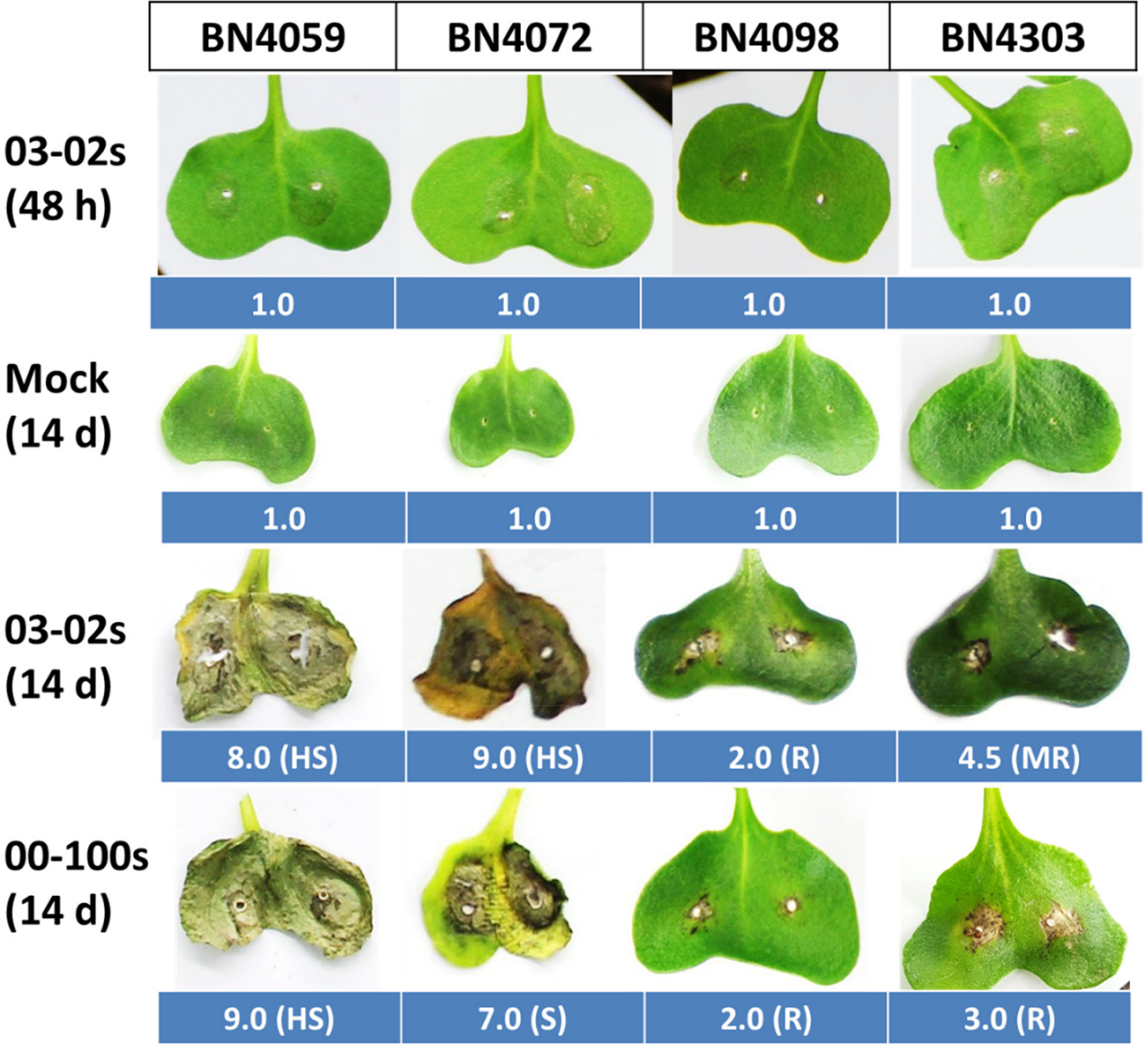
Cotyledon infection symptoms in four cabbage lines BN4059, BN4072, BN4098 and BN4303 at 14 days after inoculation in response to two isolates of *Leptosphaeria maculans* 03-02s and 00-100s. Center of each cotyledon was inoculated with 10 µl spores with a concentration of 2.0 × 10^7^spores µl^−1^. Infected leaves were scored at 14 days after inoculation on a 1–9 visual scoring scale where a lower score represents less infection and a higher score represents severe infection. Values adjacent to each cotyledon represent median score of ten observations. BN4059 and BN4072 are the susceptible lines whereas BN4098 and BN4303 are the resistant lines (Robin et al., 2017b). R, resistant; MR, moderately resistant; S, susceptible and HS, highly susceptible.

Inoculated cotyledon of four cabbage plants resulted in both hypersensitive and susceptible disease reactions. The cabbage line BN4098 showed complete resistance against both isolates, 03-02s and 00-100s, with the lowest median value of visual score (**Table 2**, **Figure 2**). BN4303 showed moderate resistance against isolate 03-02s and resistance against 00-100s (**Table 2**, **Figure 2**). Other two genotypes BN4059 and BN4072 showed susceptible disease reactions against both isolates with a high median score between 7 and 9 (**Table 2**, **Figure 2**).

**Table 2:** Resistance scoring of four cabbage lines at seedling stage against two *L. maculans* isolates ‘03-02s’ and ‘00-100s’ 14-day post-inoculation.

Line	*L. maculans* isolates					
	03-02s			00-100s		
	Score	Score range	Interaction	Score	Score range	Interaction
BN4059	8.0	7—9	HS	9.0	8—9	HS
BN4072	9.0	6—8	HS	7.0	6—8	S
BN4098	2.0	1—3	R	2.0	2—3	R
BN4303	4.5	3—5	MR	3.0	2—4	R

Each score is the median of ten observations. R, resistant; MR, moderately resistant; S, susceptible and HS, highly susceptible.

### Expression Changes in Aliphatic Glucosinolate Biosynthesis Genes in Cabbage Lines With *L. maculans* Inoculation

**Figure 3:**
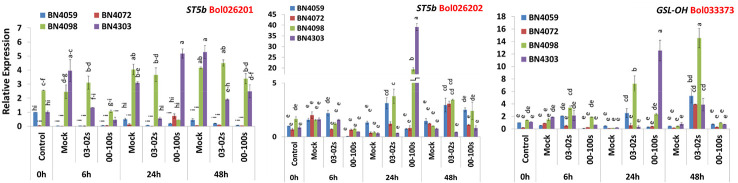
Expression levels of three aliphatic glucosinolate biosynthesis genes in leaf samples from cabbage lines. The lines BN4059 and BN4072 are susceptible and BN4098 and BN4303 are resistant to *Leptosphaeria maculans* (03-02s and 00-100s) inoculation at the seedling stage. Vertical bars represent standard error. Different letters indicate statistically significant differences between genotype × treatment combinations. Red letters represent gene accessions.

**Figure 4:**
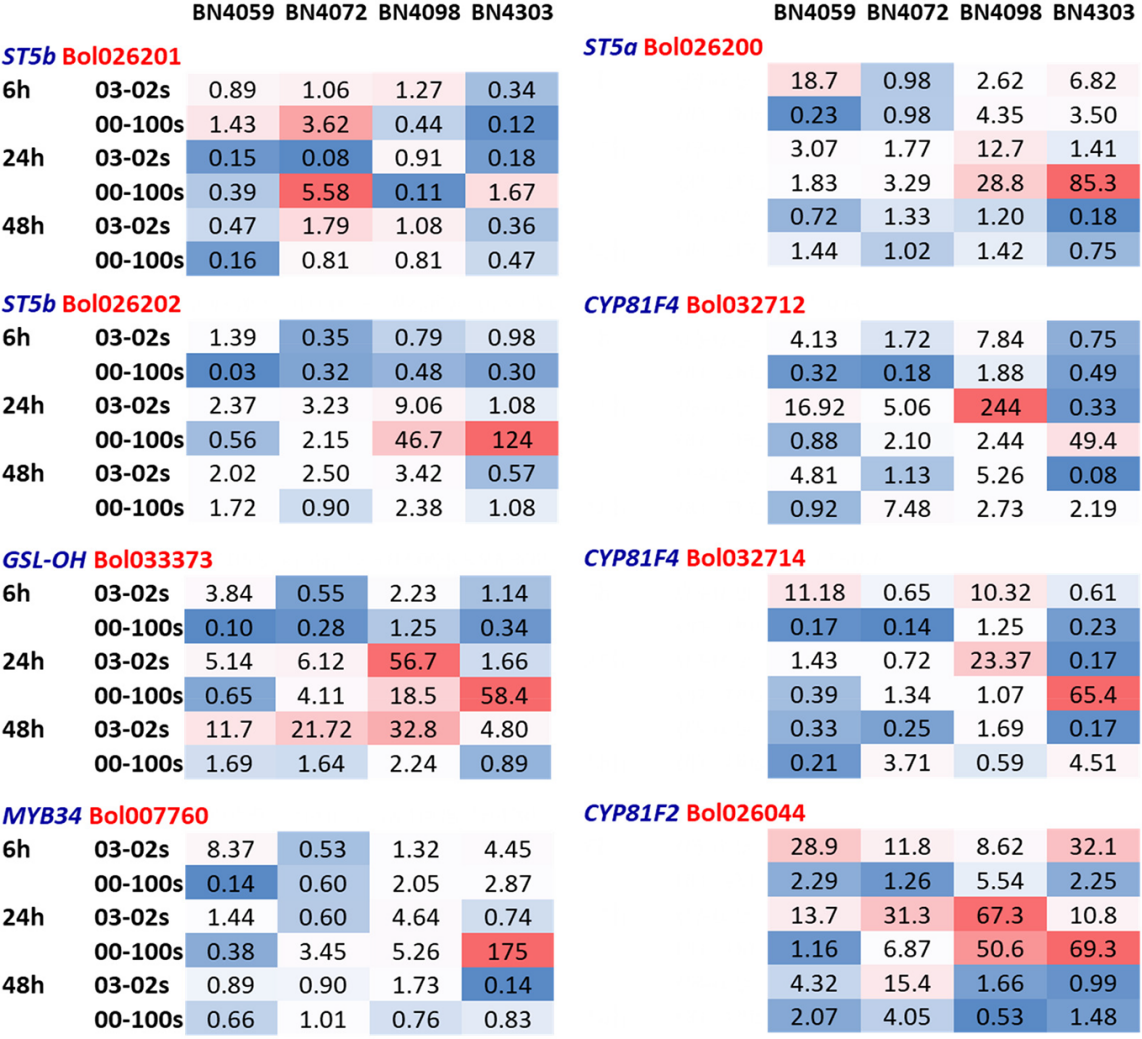
Heatmap showing fold-changes in relative expression of glucosinolate biosynthesis genes compared to respective mock treatment at each timepoint-isolate combination. The cabbage lines BN4059 and BN4072 are susceptible and BN4098 and BN4303 are resistant to *Leptosphaeria maculans* (03-02s and 00-100s) inoculation at the seedling stage. *ST5b* and *GSL-OH* are aliphatic glucosinolate biosynthesis genes. *MYB34* is an indolic transcription factor. *ST5a* and *CYP81F* are the indolic biosynthesis genes.

Three genes related to aliphatic glucosinolate biosynthesis—*ST5b* accessions Bol026201 and Bol026202, *GSL-OH* accession Bol033373 showed variable expression in four cabbage lines, time-points and treatments (**Figure 3**, **Table S1**). Expression level of Bol026201 gene was consistently lower in two susceptible lines BN4059 and BN4072 compared to two resistant lines BN4098 and BN4303 (**Figure 3**). The Bol026201 gene significantly exhibited 1.27-fold increase in expression and 0.18-fold decrease in expression in resistant line BN4303 at 24 h after inoculation with 00-100s and 03-02s isolates, respectively, compared to respective mock-treated plants (**Figure 3**). In the same line, this gene accounted for decreased expression by 0.36 and 0.47-fold at 48 h after inoculation with 03-02s and 00-100s, respectively, compared to mock-treated plants (**Figures 3** and **4**). The Bol026201 gene showed 0.11-fold decrease in expression in another resistant line BN4098 at 24 h after inoculation with 00-100s compared to mock-treated plants (**Figures 3** and **4**). The *ST5b-Bol026202* gene showed 2.37-fold increase in expression in susceptible line BN4059 at 24 h after inoculation with 03-02s compared to mock-treated plants (**Figures 3** and **4**). This gene exhibited 9.06- and 46.7-fold increase in expression in resistant line BN4098 in response to 03-02s and 00-100s isolates, respectively, at 24 h after inoculation compared to respective mock-treated plants (**Figures 3** and **4**). Strikingly, the gene accounted for 124-fold increase in expression upon infection of 00-100s isolate in another resistant line BN4303 at 24 h after inoculation compared to mock-treated plants (**Figures 3** and **4**). *ST5b-Bol026202* gene also showed 2.02-, 2.5- and 3.42-fold increase in expression at 48 h after inoculation with 03-02s isolate in cabbage lines BN4059, BN4072 and BN4098, respectively compared to respective mock-treated plants (**Figures 3** and **4**). *GSL-OH* accession Bol033373 showed 2.23-fold increase in expression in BN4098 at 6 h after inoculation with 03-02s isolate compared to mock-treated samples (**Figures 3** and **4**). This gene exhibited 5.4- and 56.7-fold upregulation in susceptible line BN4059 and resistant line BN4098, respectively; at 24 h after inoculation with 03-02s isolate compared to mock-treated samples (**Figures 3** and **4**). The Bol033373 gene also showed 11.7-, 21.7-, 32.8- and 4.8-fold increase in expression at 48 h after inoculation with 03-02s isolate in BN4059, BN4072, BN4098 and BN4303 cabbage lines, respectively compared to mock-treated samples (**Figures 3** and **4**).

### Expression Changes in Indolic Glucosinolate Biosynthesis Genes in Cabbage Lines With *L. maculans* Inoculation

**Figure 5:**
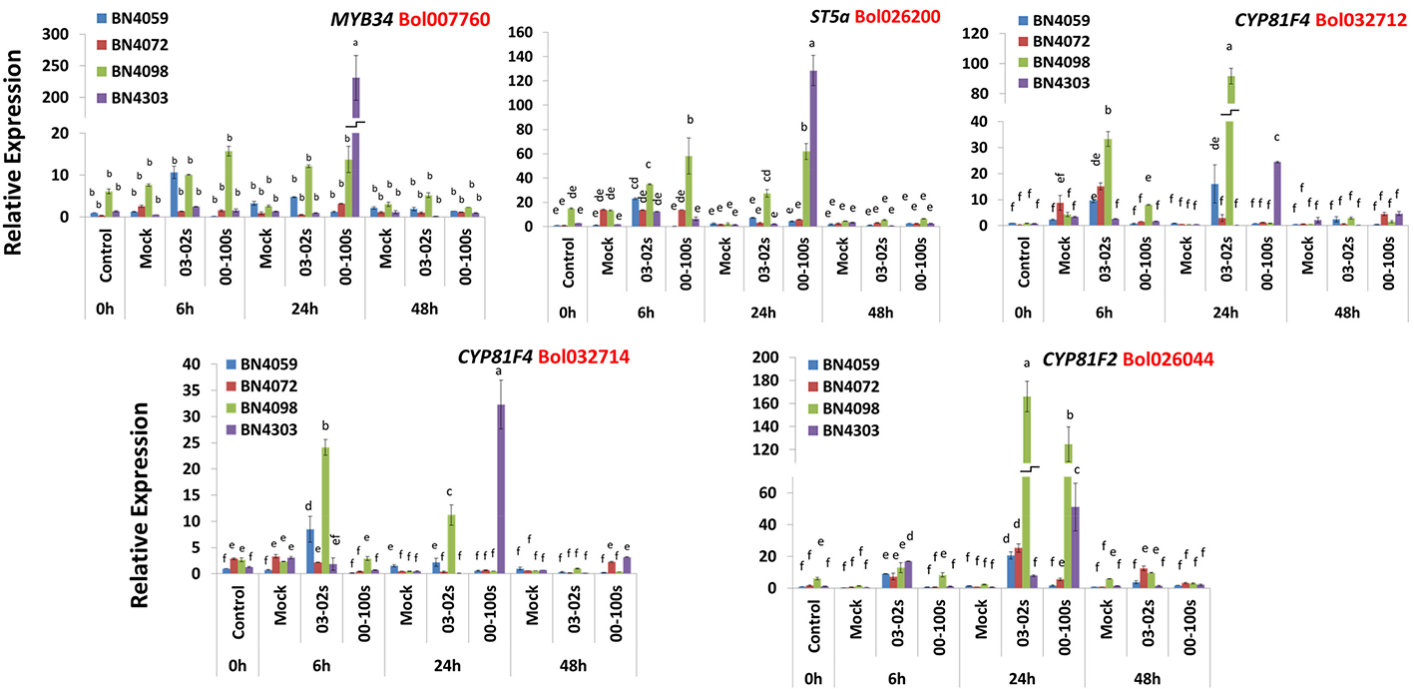
Expression levels of three indolic glucosinolate biosynthesis genes in leaf samples from cabbage lines. The cabbage lines BN4059 and BN4072 are susceptible and BN4098 and BN4303 are resistant to *Leptosphaeria maculans* (03-02s and 00-100s) inoculation at the seedling stage. Vertical bars represent standard error. Different letters indicate statistically significant differences between genotype × treatment combinations. Red letters represent gene accessions.

Like aliphatic glucosinolate biosynthesis genes, the indolic glucosinolate biosynthesis genes also showed genotypic, treatment and genotype × treatment interaction (**Table S1**). The Bol007760 accession of *MYB34* transcription factor exhibited 175-fold higher expression in resistant line BN4303 compared to mock-treated samples at 24 h after inoculation with 00-100s isolate compared to mock-treated plants (**Figures 4** and **5**). The *ST5a-Bol026200* gene accounted for 18.7-fold increase in expression in susceptible line BN4059 at 6 h after inoculation with 03-02s isolate compared to mock-treated samples (**Figures 4** and **5**). In resistant line BN4098, this gene showed 2.62- and 4.35-fold enhanced expression compared to mock-treated plants at 6 h after inoculation with 03-02s and 00-100s isolates, respectively (**Figures 4** and **5**). In the same line, BN4098, the gene exhibited 12.7- and 28.8-fold increase in expression at 24 h after inoculation with 03-02s and 00-100s isolates, respectively compared to mock-treated samples (**Figures 4** and **5**). In another resistant line, BN4303 this gene showed 85.3-fold increase in expression at 24 h after inoculation with 00-100s isolate compared to respective mock-treated plants (**Figures 4** and **5**). *CYP81F4*-*Bol032712*, exhibited 7.84- and 1.88-fold increase in expression in response to 03-02s and 00-100s isolates, respectively, at 6 h after inoculation compared to mock-treated plants (**Figures 4** and **5**). At 24 h after inoculation with 03-02s isolate, the susceptible line BN4059 showed 16.9-fold increase in expression compared to mock-treated plants (**Figures 4** and **5**). This gene exhibited strikingly higher expression, 244-fold in resistant line BN4098 and 49.4-fold in another resistant line BN4303, at 24 h after inoculation with 03-02s and 00-100s isolates, respectively compared to mock-treated plants (**Figures 4** and **5**). *CYP81F4*- *Bol032714* gene showed 11.2- and 10.3-fold increase in expression in BN4059 and BN4098 cabbage lines at 6 h after inoculation with 03-02s isolate compared to mock-treated samples (**Figures 4** and **5**). In resistant cabbage line BN4098, the gene accounted for 23.4-fold increase in expression at 24 h after inoculation with 03-02s isolate compared to mock-treated samples (**Figures 4** and **5**). In another resistant line BN4303, this gene showed 65.4-fold induced expression in response to 00-100s isolate compared to mock-treated plants (**Figures 4** and **5**). This gene also showed 3.71- and 4.51-fold increase in expression in BN4072 and BN4303 cabbage lines, respectively at 48 h after inoculation with 00-100s isolate compared to mock-treated plants (**Figures 4** and **5**). *CYP81F2*-*Bol026044* gene showed 28.9-, 11.8-, 8.6- and 32.1-fold increase in expression in response to 03-02s isolate in cabbage lines BN4059, BN4072, BN4098 and BN4303, respectively, at 6 h after inoculation compared to mock-treated samples (**Figures 4** and **5**). In resistant line BN4098, this gene also showed 5.54-fold increase in expression at 6 h after inoculation with 00-100s isolate compared to mock-treated plants (**Figures 4** and **5**). This gene showed increase in expression at 24 h after infection in two resistant lines only. *CYP81F2*- *Bol026044* accounted for 50.6- and 69.3-fold increase in expression in resistant lines BN4098 and BN4303, respectively at 24 h after inoculation with 00-100s isolate compared to mock-treated samples (**Figures 4** and **5**). This gene also showed 31.3- and 67.3-fold increase in expression at 24 h after inoculation with 03-02s isolate in BN4072 and BN4098 lines, respectively compared to mock-treated plants (**Figures 4** and **5**).

### Glucosinolate Accumulation Differs in Cabbage Inbred Lines

**Figure 6:**
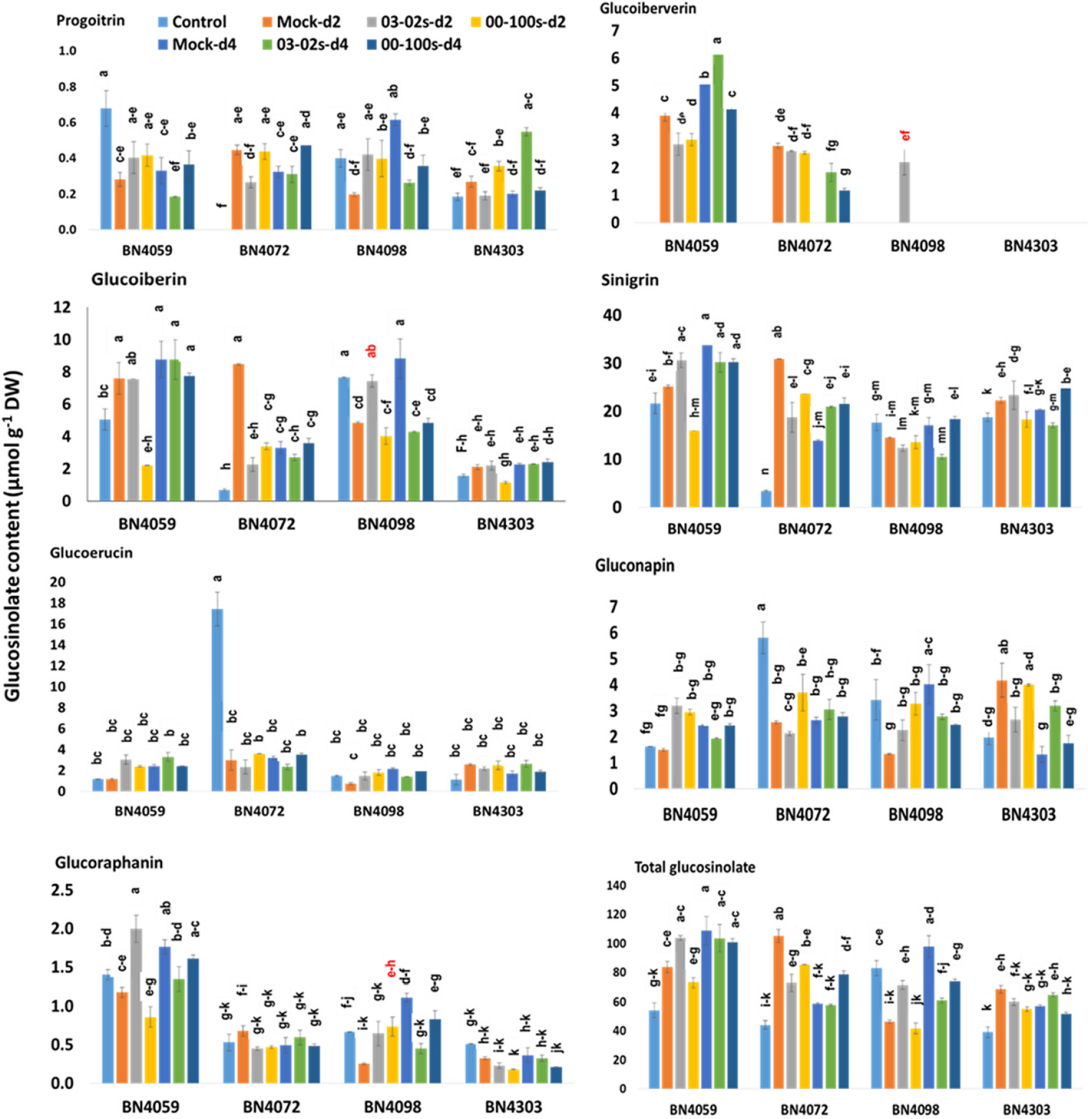
Aliphatic and total glucosinolate contents of leaf samples from four cabbage lines altered due to *Leptosphaeria maculans* at day 2 (d2) and day 4 (d4) after inoculation. The cabbage lines BN4059 and BN4072 are susceptible and BN4098 and BN4303 are resistant to *L. maculans* (03-02s and 00-100s) inoculation at the seedling stage. The mean of three biological replicates is presented. Vertical bars represent standard error. Different letters indicate statistically significant differences between genotype × treatment combinations. Red letters indicate increased glucosinolate content in resistant lines in response to *L. maculans* infection compared to mock treatment.

**Figure 7:**
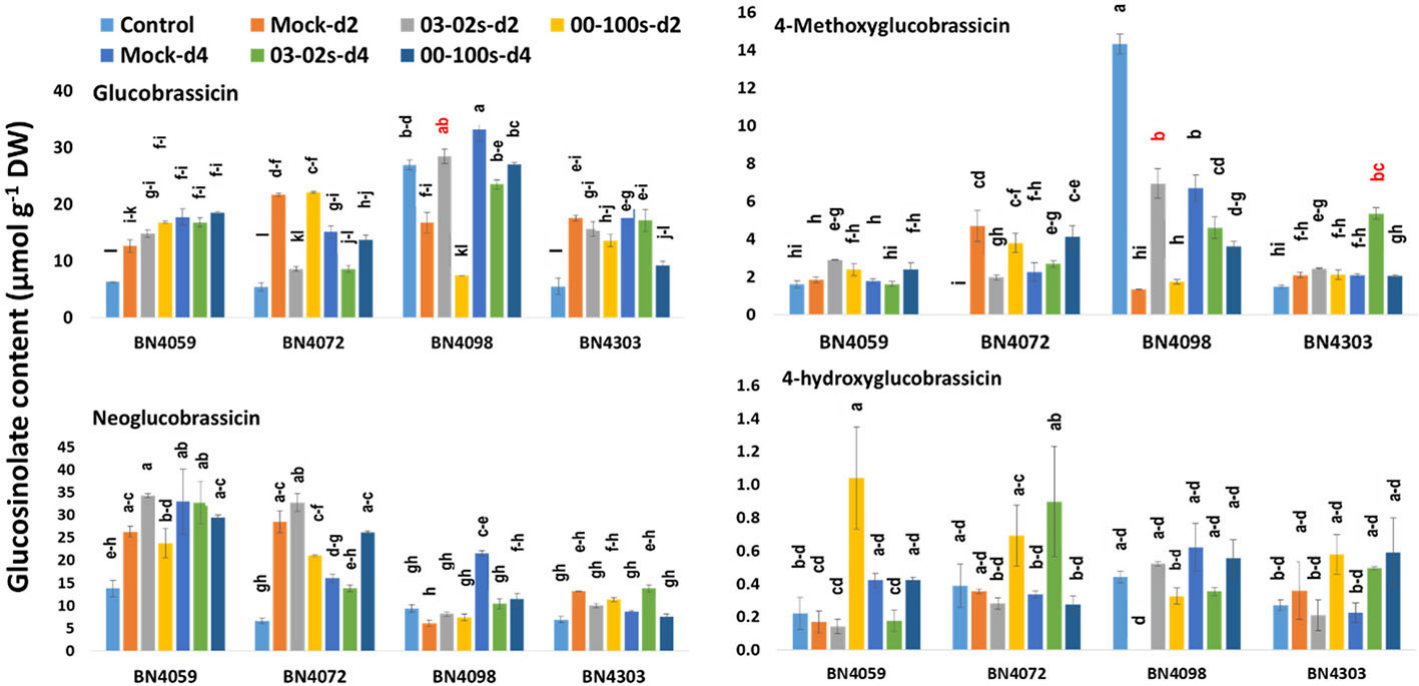
Indolic glucosinolate contents of leaf samples from four cabbage lines altered due to *Leptosphaeria maculans* at day 2 (d2) and day 4 (d4) after inoculation. The cabbage lines BN4059 and BN4072 are susceptible and BN4098 and BN4303 are resistant to *L. maculans* (03-02s and 00-100s) inoculation at the seedling stage. The mean of three biological replicates is presented. Vertical bars represent standard error. Different letters indicate statistically significant differences between genotype × treatment combinations. Red letters indicate increased glucosinolate content in resistant lines in response to *L. maculans* infection compared to mock treatment.

In untreated plants, contents of aliphatic, indolic and total glucosinolates differed among four cabbage lines (**Figures 6** and **7**, **Table S2**). Constitutively, the susceptible cabbage line BN4059 recorded the highest levels of aliphatic progoitrin (PRO), glucoiberverin (GIV), sinigrin (SIN), glucoraphanin (GRA) and indolic neoglucobrassicin (NGBS) (**Figures 6** and **7**). Another susceptible line BN4072, estimated the highest contents of aliphatic glucoerucin (GER) and gluconapin (GNA) (**Figure 6**). By contrast, the resistant cabbage line BN4098 accumulated the highest levels of aliphatic glucoiberin (GIB), total glucosinolate and indolic glucobrassicin (GBS), 4-methoxyglucobrassicin (MGBS) and 4-hydroxy-glucobrassicin (HGBS) (**Figures 6** and **7**). The untreated control plants of cabbage line BN4072 detected no PRO, GIV and MGBS but this cabbage line accumulated 14-fold higher content of GER compared to average of other three lines (**Figures 6** and **7**). Two resistant lines BN4098 and BN4303 also detected no GIV in untreated control samples (**Figure 6**).

### Altered Glucosinolate Biosynthesis in Cabbage Lines With *L. maculans* Inoculation

Leaf inoculation of resistant and susceptible cabbage lines, with either of two *L. maculans* fungal isolates responsible for blackleg disease, altered accumulation of both total glucosinolate and individual glucosinolate components in the leaf samples. The content of aliphatic PRO decreased by 0.42-fold in resistant cabbage line BN4098 but that increased by 2.73-fold in another resistant line BN4303 compared to respective mock-treated samples at four days after inoculation (d4) with 03-02s isolate (**Figure 6**). The content of GIV showed inconsistent pattern of accumulation in four cabbage lines. The resistant line BN4303 detected GIV neither in treated nor in non-treated samples (**Figure 6**). Another resistant line BN4098 detected 2.2 µmol g^−1^ DW of GIV only in the treated plants at two days after inoculation (d2) with 03-02s isolate (**Figure 6**). In susceptible cabbage line BN4059, the content of GIV decreased by 0.77- and 0.78-fold at both d2 and d4, respectively, after inoculation with 00-100s isolate compared to mock-treated samples (**Figure 6**). In the same line, the content of GIV decreased by 0.73-fold at d2 but increased by 1.22-fold at d4 after inoculation with 03-02s isolate compared to mock-treated samples (**Figure 6**). In another susceptible line BN4072, the contents of GIV were 1.83 and 1.18 µmol g^−1^ DW at d4 after inoculation with 03-02s and 00-100s isolates, respectively, whereas both mock-treated plants at d4 and untreated control plants detected no GIV (**Figure 6**). The content of GIB in susceptible lines decreased by 0.29-, 0.4- and 0.4- fold in BN4059 × 00-100s, BN4072 × 03-02s and BN4072 × 00-100s interactions, respectively at d2 compared to mock-treated plants (**Figure 6**). In resistant line BN4098, content of GIB increased by 1.53-fold at d2 in response to 03-02s compared to mock-treated plants but that was decreased by 0.48- and 0.55-fold at d4 after inoculation with 03-02s and 00-100s isolates, respectively (**Figure 6**). The content of SIN decreased in two susceptible lines in response to fungal inoculation. In cabbage line BN4059, the content of SIN decreased by 0.63-fold at d2 in response to 00-100s compared to mock-treated plants (**Figure 6**). SIN content in cabbage line BN4072 decreased by 0.61- and 0.77-fold at d2 in response to 03-02s and 00-100s isolates, respectively, compared to mock-treated plants (**Figure 6**). The content of GRA was generally higher in susceptible line BN4059 at both d2 and d4 in response to both treatments (**Figure 6**). In cabbage line BN4059, the content of GRA increased by 1.7-fold at d2 after inoculation with 03-02s isolate compared to mock-treated plants (**Figure 6**). In resistant line BN4098, the content of GRA increased by 2.93-fold at d2 and decreased by 0.4-fold at d4 after inoculation with 00-100s and 03-02s isolates, respectively, compared to mock-treated plants (**Figure 6**).

Among the indolic glucosinolates, the content of GBS increased by 1.7-fold in resistant line BN4098 at d2 in response to 03-02s compared to mock-treated plants. In the same cabbage line, GBS accumulation decreased by 0.71- and 0.81-fold at d4 after inoculation with 03-02s and 00-100s, respectively, compared to mock-treated plants (**Figure 7**). In another resistant cabbage line BN4303, GBS accumulation decreased by 0.48-fold at d4 after inoculation with 00-100s compared to mock-treated plants (**Figure 7**). In susceptible cabbage line BN4072, the content of GBS decreased by 0.4- and 0.57-fold at d2 and d4, respectively, after inoculation with 03-02s isolate (**Figure 7**). The content of MGBS increased strikingly by 5.27-fold in resistant cabbage line BN4098 at d2 after inoculation with 03-02s isolate compared to mock-treated plants (**Figure 7**). In the same line, MGBS accumulation decreased by 0.69- and 0.54-fold at d4 after inoculation with 03-02s and 00-100s isolates, respectively, compared to mock-treated plants (**Figure 7**). MGBS accumulation also increased by 2.56-fold in another resistant cabbage line BN4303 at d4 after inoculation with 00-100s isolate (**Figure 7**). In the susceptible cabbage line BN4072, the content of MGBS decreased by 0.42-fold at d2 after inoculation with 03-02s isolate and increased by 1.84-fold in response to 00-100s isolate compared to mock-treated plants (**Figure 7**). Another indolic glucosinolate, NGBS showed increased accumulation by 1.63-fold at d4 after inoculation with 00-100s compared to mock-treated plants (**Figure 7**). NGBS accumulation decreased by 0.48- and 0.53-fold at d4 after inoculation with 03-02s and 00-100s, respectively compared to mock-treated plants (**Figure 7**). The content of HGBS showed increase in accumulation only in cabbage line BN4059 at d2 after inoculation with 03-02s isolate compared to mock-treated plants (**Figure 7**).

In brief, the contents of both aliphatic and indolic glucosinolates induced in two resistant cabbage inbred lines upon infection of two *L. maculans* isolates (**Figures 6** and **7**). Contents of GIB and GIV induced in BN4098 × 03-02s interaction whereas GRA was induced in BN4098 × 00-100s interaction (**Figure 6**, **Table 3**). MGBS accumulation induced in both BN4303 and BN4098 but the GBS accumulation induced only in BN4098 cabbage line (**Table 3**).

**Table 3:** Association between enhanced expressions of glucosinolate (GSL) pathway genes, enhanced glucosinolate accumulation in resistance disease interactions.

		BN4098 × 03-02s(Resistant interaction)	BN4098 × 00-100s(Resistant interaction)	BN4303 × 03-02s(Moderately resistant interaction)	BN4303 × 00-100s(Resistant interaction)
Aliphatic	Gene	(*ST5b*-*Bol026202*)(9.06-fold, d1) *(GSL-OH*-*Bol033373*)(56.7-fold, d1)	(*ST5b*-*Bol026202*)(46.7-fold, d1) *(GSL-OH*-*Bol033373*)(18.5-fold, d1)	*GSL-OH*-*Bol033373*(4.8 fold, d2)	(*ST5b*-*Bol026202*)(124-fold, d1) *(GSL-OH*-*Bol033373*)(58.4-fold, d1)
	GSL	GIB (1.53-fold, d2) GIV (2.2 µmol g^−1^ DW, d2)	GRA (2.93-fold, d2)		
Indolic	Gene	*ST5a*-*Bol026200*(12.7-fold, d1) *CYP81F4*-*Bol032712*(244-fold, d1) *CYP81F2*-*Bol026044*(67.3-fold, d1)	*ST5a*-*Bol026200*(28.8-fold, d1) *CYP81F4*-*Bol032712*(1.88-fold, 6h) *CYP81F2*-*Bol026044*(50.6-fold, d1)	*CYP81F2*-*Bol026044*(32.1-fold, 6h)	*MYB34*-*Bol007760*(175-fold, d1) *ST5a*-*Bol026200*(85.3-fold, d1) *CYP81F4*-*Bol032712*(49.4-fold, d1) *CYP81F2*-*Bol026044*(69.3-fold, d1)
	GSL	GBS (1.7-fold, d2)MGBS (5.27-fold, d2)			MGBS (2.56-fold, d4)

d1, d2 and d4; one, two and four days after inoculation. In general, transcript levels increased at d1 and glucosinolate accumulation induced at d2 in resistant lines upon L. maculans inoculation. Blue letters highlight fold-changes and red letter represent days after inoculation.

### Association Between Expression Levels of Pathway Genes and Accumulation of Glucosinolates Considering Resistance Response of Plants

Pearson correlation analysis revealed that *ST5b-Bol026201* gene showed negative correlation with contents of aliphatic GRA, GIV and GER but *ST5b-Bol026202* gene showed positive correlation with contents of GIB, GRA and GIV (**Table 4A**). Expression analysis revealed that *ST5b-Bol026202* exhibited strikingly higher expression at 24 h after inoculation with 00-100s isolate in both resistant lines (46.7-fold in BN4098 and 124-fold in BN4303) compared to mock-treated samples (**Figure 3**). Simultaneously, the content of GRA, among these three aliphatic glucosinolates, increased by 2.93-fold in resistant line BN4098 at d2 after inoculation with 00-100s isolate (**Figure 6**). The other aliphatic gene *GSL-OH* accession Bol033373 showed positive correlation only with the GIB content (**Table 4A**). The expression levels of this *GSL-OH* gene was highly increased in the resistant line BN4098 by 56.7- and 32.8-fold at 24 h and 48 h, respectively, after inoculation with 03-02s isolate compared to mock-treated samples (**Figure 3**). Concurrently, aliphatic GIB accumulation was increased only in the resistant line BN4098 by 1.53-fold at d2 in response to 03-02s isolate compared to mock-treated samples (**Figure 6**).

**Table 4:** Pearson correlation coefficient between glucosinolate contents (aliphatic and indolic) and relative expression level of genes.

A) Aliphatic Glucosinolates					
	***ST5b-Bol026201***		***ST5b-Bol026202***		***GSL-OH-Bol033373***
Glucoiberin	0.018 0.901		**0.361** **0.012**		**0.303** **0.036**
Progoitrin	−0.044 0.766		0.237 0.104		0.125 0.397
Glucoraphanin	**−0.383** **0.007**		**0.380** **0.008**		0.133 0.369
Sinigrin	−0.254 0.082		−0.038 0.797		−0.092 0.533
Gluconapin	−0.020 0.893		−0.199 0.175		−0.160 0.279
Glucoiberverin	**−0.506** **<0.001**		**0.531** **<0.001**		0.207 0.158
Glucoerucin	**−0.305** **0.035**		−0.219 0.134		−0.156 0.291
**B) Indolic Glucosinolates**					
	***MYB34-Bol007760***	***ST5a-Bol026200***	***CYP81F4-Bol032712***	***CYP81F4-Bol032714***	***CYP81F2-Bol026044***
4-hydroxyglucobrassicin	0.013 0.932	0.076 0.610	0.264 0.070	0.220 0.134	−0.043 0.771
Glucobrassicin	**0.613** **<0.001**	**0.483** **0.001**	**0.304** **0.036**	0.083 0.577	0.270 0.063
4-Methoxyglucobrassicin	**0.783** **<0.001**	**0.838** **<0.001**	0.079 0.594	0.276 0.058	**0.319** **0.027**
Neoglucobrassicin	−0.233 0.112	−**0.287** **0.048**	0.004 0.980	−**0.372** **0.009**	0.116 0.432

Bold letters represent significant correlation. Cell Contents: Pearson correlation and P-Value.

GBS accumulation was positively correlated with expression of *MYB34-Bol007760*, *ST5a-Bol026200* and *CYP81F4-Bol032712* (**Table 4B**). While the content of GBS increased by 1.7-fold in resistant line BN4098 at d2 after inoculation with 03-02s compared to mock-treated plants, the expression level of *CYP81F4-Bol032712* gene increased by 244-fold in BN4098 × 03-02s at 24 h after inoculation (**Figures 5** and **7**). Expression levels of *MYB34-Bol007760*, *ST5a-Bol026200* and *CYP81F2-Bol026044* were positively associated with MGBS accumulation (**Table 4B**). The expression levels of *MYB34-Bol007760*, *ST5a-Bol026200* and *CYP81F2-Bol026044* genes increased by 175-, 85.3- and 69.3-fold, respectively, in resistant cabbage line BN4303 at 24 h after inoculation with 00-100s isolate compared to mock-treated plants (**Figure 5**). Expression of *CYP81F2-Bol026044* gene also increased by 67.3-fold in another resistant line BN4098 at 24 h after inoculation with 03-02s isolate compared to mock-treated plants (**Figure 5**). Simultaneously, the content of MGBS increased by 5.27- and 2.56-fold in BN4098 × 03-02s interaction at d2 and BN4303 × 00-100s interaction at d4, respectively, compared to mock-treated plants (**Figure 7**). Expression levels of *ST5a-Bol026200* and *CYP81F4-Bol032714* were negatively correlated with contents of NGBS (**Table 4B**).

**Figure 8:**
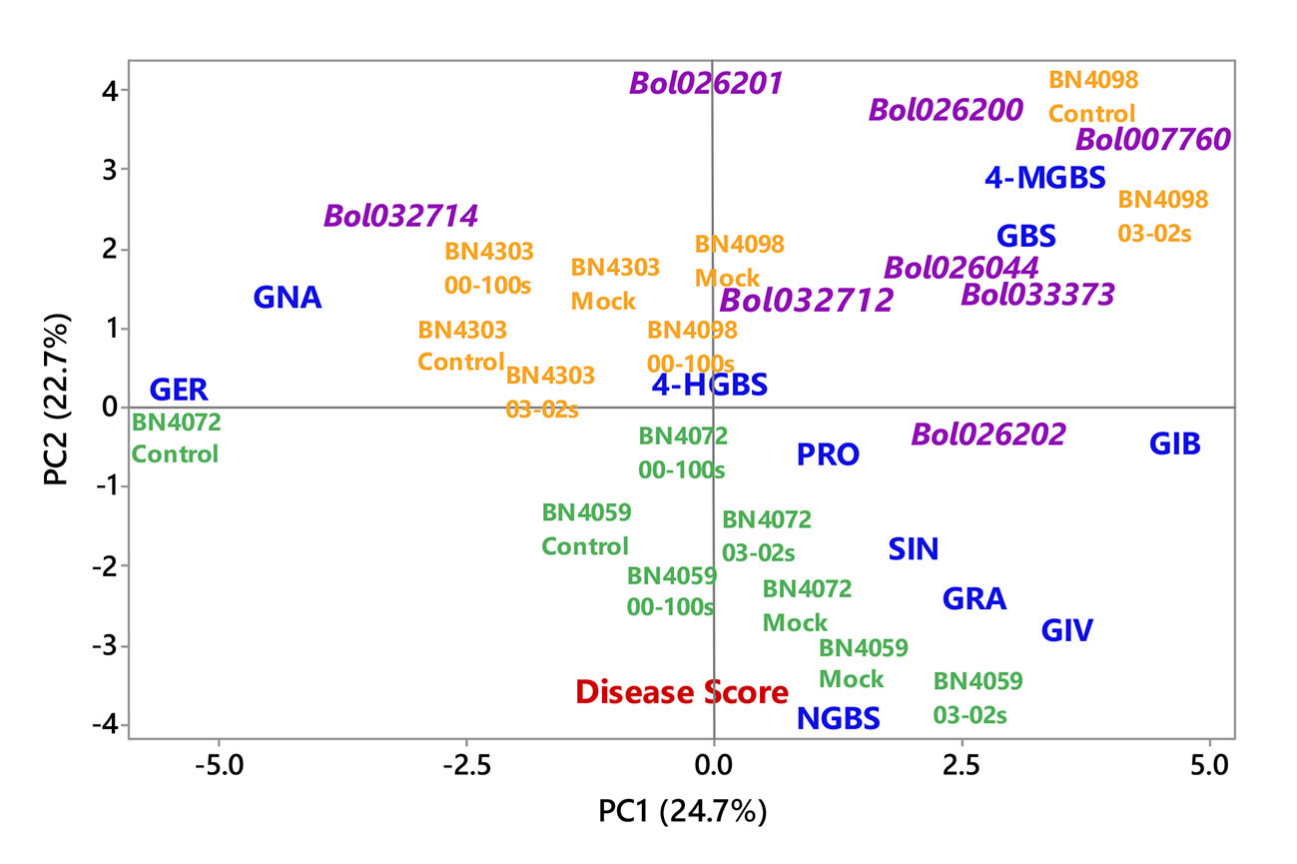
Biplot of individual glucosinolate contents of leaf samples (blue color), disease scoring index (red color) and relative expression of glucosinolate biosynthesis genes (magenta color) from four cabbage lines altered due to *Leptosphaeria maculans* inoculation at two days after inoculation. The cabbage lines BN4059 and BN4072 are susceptible (green color) and BN4098 and BN4303 are resistant (orange colour) to *L. maculans* (03-02s and 00-100s) inoculation at the seedling stage. Glucosinolate (GSL) components: GER, glucoerucin; GNA, gluconapin; GIB, glucoiberin; PRO, progoitrin; GRA, glucoraphanin; SIN, sinigrin; GIV, glucoiberverin; GBS, glucobrassicin; 4-MGBS, 4-methoxyglucobrassicin; NGBS, neoglucobrassicin; 4-HGBS, 4-hydroxyglucobrassicin.

First two principal components, PC1 and PC2, explained 24.7 and 22.7% data variation, respectively. PC1 separated the contents of MGBS, GBS, GIB, GIV and GRA for their positive coefficients from the contents of GER and GNA for their negative coefficients (**Figure 8**). The enhanced accumulation of MGBS, GBS, GIB, GIV and GRA after the inoculation of fungal isolates was the feature of resistant cabbage lines (**Figures 6** and **7**). PC1 also separated the expression levels of *MYB34-Bol007760*, *ST5a-Bol026200*, *CYP81F4-Bol032712*, *CYP81F2-Bol026044*, *GSL-OH*-*Bol033373* and *ST5b-Bol026202* for their positive coefficients from the expression levels of *ST5b-Bol026201* and *CYP81F4-Bol032714* for their negative coefficients (**Figure 8**). Increased expression of *MYB34-Bol007760*, *ST5a-Bol026200*, *CYP81F4-Bol032712*, *CYP81F2-Bol026044*, *GSL-OH*- *Bol033373* and *ST5b-Bol026202* genes enhanced accumulation of indolic and aliphatic glucosinolates in the resistant cabbage lines BN4098 and BN4303 (**Tables 4A, B**, **Figures 6** and **7**). PC2 separated two susceptible lines BN4059 and BN4072 for their high sensitivity to blackleg disease (disease scores) from two resistant lines BN4098 and BN4303 for their comparatively lower disease scores (**Figure 8**).

Principal component analysis showed a strong association between induced glucosinolate accumulation, expression level of genes and blackleg disease response indicating that pathogen-induced glucosinolate accumulation has a strong influence on blackleg disease resistance in cabbage plants (**Figure 8**). Glucosinolate contents and expression levels of glucosinolate biosynthetic genes changed dramatically in response to two *L. maculans* isolates 03-02s and 00-100s in two resistant lines (**Table 3**). Alteration of glucosinolate accumulation and expression levels of biosynthesis genes occurred concomitantly in two resistant cabbage inbred lines BN4098 and BN4303 (**Table 3**). Most of the glucosinolate biosynthetic genes were highly transcribed at 24 h after inoculation of *L. maculans* isolates (**Table 3**). As a resultant effect the contents of glucosinolates were significantly changed in resistant lines at two days after inoculation, in most cases (**Table 3**). Only the contents of MGBS showed increase in accumulation at d4 after inoculation in BN4303 × 00-100s interaction (**Figure 7**).

## Discussion

### Glucosinolate Accumulation and Expression of Relevant Genes Were Associated With Blackleg Disease Resistance at the Seedling Stage

In our previous study we measured glucosinolate accumulation only at one time-point—at d4 after inoculation (Robin et al., 2017a) but here we have found that glucosinolate accumulation dynamically changed between two and four days after *L. maculans* infection (**Figures 6** and **7**). The selection of single time-point (d4) in Robin et al. (2017a) was based on some previous studies that looked at effect of exogenous application of methyl jasmonate and salicylic acid on glucosinolate accumulation (Ku et al., 2013a; Ku et al., 2013b; Yi et al., 2016). Induced glucosinolate accumulation at two days after inoculation of *L. maculans* indicated an immediate response of inoculated plants to tackle the disease. More interestingly, the major changes in pathogen-induced glucosinolate accumulation were followed by increasing transcript accumulation at the previous day (**Table 3**).

Moreover, an increase of a particular glucosinolate at a single time-point was not associated with resistance. In general, accumulation of individual glucosinolate components decreased in susceptible cabbage lines—BN4059 and BN4072 except a few cases (**Figures 6** and **7**). GIV in BN4059 × 03-02s at d4, GRA of in BN4059 × 03-02s at d2 and MGBS in BN4072 × 00-100s in d2 showed increase in contents in response to pathogen inoculation but their contents decreased in other time-points (**Figures 6** and **7**). This disparity in susceptible lines and pathogen-induced accumulation in resistant lines indicated that resistance of cabbage inbred lines to *L. maculans* follow genotype- and isolate-specific manner.

### Pathogen-Induced Glucosinolate Accumulation and Hypersensitivity Response to *L. maculans* Differ Between Seedling and Adult Stages of Cabbage Plants

In adult cabbage plants, the contents of aliphatic GER and indolic NGBS were induced in resistant cabbage lines in addition to GIV, GBS and MGBS but aliphatic GRA was not induced (Robin et al., 2017a). These results indicated that glucosinolate mediated resistance in cabbage plants differs between seedling and adult stages. In consistence with variable accumulation of individual glucosinolate compound between seedling and adult stages, the hypersensitivity response also differed. The cabbage inbred line BN4098 showed susceptibility reaction against 03-02s isolate and moderately resistance against 00-100s isolate at the adult stage but in this study this line showed complete resistance at the seedling stage (**Figure 2**, Robin et al., 2017a). Another resistant line BN4303 exhibited moderate resistance against 00-100s at the seedling stage but that interaction was complete resistance at the adult stage (**Figure 2**, Robin et al., 2017a). The disparity in hypersensitivity response between seedling stage and adult stage against blackleg disease is known for quite a long since these two stages have different genetic control (Ballinger and Salisbury, 1996; Larkan et al., 2016) and the resistance of adult plants may not be race-specific (Rimmer, 2006). Moreover, recent findings believe that hypersensitivity response of plants requires complex interaction between proteins related to innate immunity in plants and those related to glucosinolate biosynthesis (Nintemann et al., 2017).

### Accumulation of GIV and Other Aliphatic Glucosinolates Differs in Resistant and Susceptible Lines

In 3-month old cabbage plants accumulation of GIV induced in BN4303 in response to both 03-02s and 00-100s isolates at d4 after inoculation and two resistant lines accumulated GIV in both treated and non-treated samples (Robin et al., 2017a). In this study, the content of GIV showed inconsistent pattern of accumulation among the tested cabbage inbred lines. No GIV was detected in two resistant plants, except BN4098 × 03-02s at d2, whereas two susceptible lines accumulated abundant GIV in both treated and non-treated plants (**Figure 6**). This results supported the fact that glucosinolate mediated resistance in cabbage plants differs between seedling and adult stages. In recent studies, resistant cabbage plants inoculated with *S. sclerotiorum* and *M. brassicicola* showed increased accumulation of GIV (Abuyusuf et al., 2018a; Abuyusuf et al., 2018b) indicating its importance in resistance against diverse fungal infection.

GRA is the aliphatic glucosinolate was not induced in any previous studies after inoculation with diverse fungal species including *L. maculans*, *S. sclerotiorum*, *M. brassicicola* and *Albugo candida* in Brassicaceae family (Singh et al., 2015; Robin et al., 2017a; Abuyusuf et al., 2018a; Abuyusuf et al., 2018b). Thus, this compound was induced only at the seedling stage rather than adult stage (Robin et al., 2017a). In resistant line BN4098, content of GIB increased by 1.53-fold at d2 upon infection of 03-02s compared to mock-treated plants but in all other combination it was decreased (**Figure 6**). GIB and PRO were induced significantly in leaves of broccoli and roots of Indian mustard, compared to the control, after inoculation with *A. candida* (Singh et al., 2015). SIN have a potential role in suppressing the growth of *S. sclerotiorum* fungus and reducing disease severity in kale (Madloo et al., 2019). These results indicated GIV and other aliphatic glucosinolates have vital role in plant-microbe interactions in Brassicaceae family.

### Role of Indolic GBS and MGBS in Blackleg Disease Resistance at the Seedling Stage

Our results indicated that pathogen-induced MGBS accumulation can be anti-oxidative to both *L. maculans* isolates since accumulation of MGBS at the seedling stage was greatly induced by 5.27- and 2.56-fold in two resistant interactions in two different cabbage lines—BN4098 × 03-02s at d2 and BN4303 × 00-100s at d4, respectively (**Table 3**, **Figure 7**). In the sensitive cabbage line, the content of MGBS decreased (**Figure 7**) indicating that indolic MGBS has a pivotal role in blackleg resistance in *B. oleracea*.

The accumulation of GBS induced in BN4098 × 03-02s at d2 by 1.7-fold, similar to our previous study (Robin et al., 2017a). In the adult plants, however, the induced accumulation of GBS was observed in both resistant lines against 00-100s isolate that showing difference in pathogen-induced GBS accumulation in cabbage seedlings. Role of indolic glucosinolates in offering resistance against a number of fungal species is established (Li et al., 1999b; Robin et al., 2017a; Abuyusuf et al., 2018a; Abuyusuf et al., 2018b). In Chinese and European cultivars of *B. napus*, the accumulation of indolic glucosinolate was induced upon inoculation of *S. sclerotiorum* (Li et al., 1999b). Notably, indolic glucosinolate alone can provide resistance to both nectrotrophic and hemibiotrophic pathogens (Sanchez-Vallet et al., 2010; Hiruma et al., 2013; Frerigmann et al., 2016; Wu et al., 2016). Other than the anti-fungal properties, indolic GBS and aromatic glucosinolate inhibits infection by bacteria, e.g., infection by *Xanthomonas campestris* pv. *campestris* in kale (Madloo et al., 2019); protists, e.g., infection by *Plasmodiophora brassicae* in *B. napus* (Xu et al., 2018) and insects, e.g., infestation by Diamond backmoth in cabbage (Robin et al., 2017c).

### Increased Transcription of *ST5b-Bol026202* Gene Led to Increased Accumulation of GRA, GIB and GIV in Resistant Cabbage Line

ST5b is a sulfotransferase protein involved in aliphatic glucosinolate pathway. In our previous with adult cabbage plants, the expression level ST5b-*Bol026202* gene was only positively associated the contents of GIV (Robin et al., 2017a) but at the seedling stage this gene was found positively correlated with accumulation of aliphatic GRA, GIB and GIV (**Table 4A**) and increase in the accumulation of these three glucosinolate compounds was associated with blackleg disease resistance. In response to *M. brassicicola*, cabbage seedlings at third leaf-stage showed positive association between expression of ST5b-*Bol026202* gene and accumulation of GIB and GIV (Abuyusuf et al., 2018b). In another study, this gene showed positive correlation only with GIV accumulation upon infection of *S. sclerotiorum* in two months old cabbage plants (Abuyusuf et al., 2018a). These results indicated that pathogen-induced transcript accumulation of ST5b-*Bol026202* gene is more pronounced at the seedling stage that alter glucosinolate accumulation.

### *MYB34* Likely Activated Higher GBS and MGBS Accumulation in Blackleg Resistant Cabbage Lines

*MYB34-Bol007760* gene showed positive correlation both GBS and MGBS in our previous study with blackleg disease infection at the adult stage similar to this study (Robin et al., 2017a). This gene also showed positive correlation with both GBS and MGBS in response to *Mycosphaerella brassicicola* infection (Abuyusuf et al., 2018a). But under the infection of *S. sclerotiorum* this gene exhibited positive correlation with NGBS only, in two months old cabbage plants (Abuyusuf et al., 2018b). The biosynthesis of indolic glucosinolates was directly regulated by the *MYB34* genes in *Arabidopsis* (Frerigmann and Gigolashvili, 2014) and *B. oleracea* (Yi et al., 2016; Robin et al., 2016). Moreover, *MYB34*, in combination with *MYB122* and *MYB51* in *Arabidopsis*, actively favour protection against *Plectosphaerella cucumerina*, where *PEN2* (*PENETRATION2*) plays a key role in enhancing the pathogen-induced expression of specific biosynthesis genes (Frerigmann et al., 2016). In a previous study, this *MYB34-Bol007760* gene was found to be induced upon elicitation with methyl jasmonate in cabbage lines, indicating pathogen-induced resistance might follow jasmonic acid signaling (Yi et al., 2016).

### Accumulation of Indolic GBS in Blackleg Resistant Cabbage Lines Is Triggered by Increased Expression of *ST5a-Bol026200* and *CYP81F4-Bol032712* Genes

Expression levels of both *ST5a-Bol026200* and *CYP81F4-Bol032712* genes showed positive correlation with GBS accumulation at both seedling and adult stage of cabbage plants (**Table 4B**, Robin et al., 2017a). GBS, has antifungal reactions in plants, exhibited increased accumulation in the resistance line, as compared to mock-treated plants (**Figure 7**). GBS accumulation was associated with upregulation of *CYP81F4* and *CYP81F2* in *S. sclerotiorum* infection (Abuyusuf et al., 2018a). Similar association was also found between GBS levels and *CYP81F2* upregulation in *B. oleracea* (Sotelo et al., 2016*)*.

### Increased Transcription of *ST5a-Bol026200* and *CYP81F2-Bol026044* Lead to Accumulation of MGBS and Is Associated Blackleg Resistance in Cabbage at the Seedlings Stage

At the 3-months old adult plants, increase in accumulation of MGBS upon *L. maculans* inoculation was associated with moderate resistance of BN4098 line against 00-100s isolate (Robin et al., 2017a) but in this study, pathogen-induced MGBS accumulation by 2–5 folds was associated with complete resistant of 28–30 days old cabbage seedlings of both resistant lines—BN4098 and BN4303 (**Table 3**, **Figure 2**). MGBS was also induced in *B. napus* after 5–8 days of infection of *L. maculans* (Wretblad and Dixelius, 2000). The pathogen-induced increase in MGBS was strongly associated with expression level of *ST5a-Bol026200* gene and moderately associated with expression level of *CYP81F2-Bol026044* (**Table 4B**). Transcript abundance of *CYP81F2* increased in both cabbage and *Arabidopsis* upon infection of several other pathogens including *Plectosphaerella cucumerina*, *Erysiphe pisi*, and *Blumeria graminis* (Bednarek et al., 2009). Thus the results of our study is consistent with a previous report indicating that both *CYP81F2* (*Bol026044*) and myrosinase PEN2, which hydrolyzes MGBS, simultaneously induce antifungal defense (Bednarek et al., 2009).

## Conclusions

The GSL profiling and expression analysis of GSL-related genes in cabbage infected by *L. maculans* reveal that glucosinolate accumulation and relevant gene expression were directly associated with blackleg resistance at the seedling stage of cabbage. This study showed that both aliphatic and indolic glucosinolates take part in resistance to blackleg disease in a genotype- and isolate-specific manner. Enhanced MGBS content against both fungal isolates, contributing to seedling resistance in two interactions- BN4098 × 03-02s and BN4303 × 00-100s --- and enhanced GBS content contributed to the resistance of BN4098 x 00-100s interaction only. Aliphatic GRA took part in resistance of BN4098 × 00-100s interaction whereas aliphatic GIB took part is resistance of BN4098 × 03-02s interaction. Aliphatic GIV accumulated in BN4098 × 03-02s interaction but *GSL-OH*-*Bol033373* and *CYP81F2*-*Bol026044* showed enhanced expression in BN4303 × 03-02s interaction. The GSLs and the corresponding genes identified in this study could improve blackleg resistance in cabbage seedlings.

## Data Availability Statement

All datasets presented in this study are included in the article/**Supplementary Material**.

## Author Contributions

I-SN, J-IP, and AR conceived of and designed the study. AR managed and inoculated the experimental plants, collected samples, prepared cDNA, performed the qPCR analysis, and prepared samples for HPLC. RL assisted with the cDNA preparation and sample preparation for HPLC analysis. AR and MA wrote the manuscript. AR critically revised the manuscript.

## Funding

This study was supported by the Center for Horticultural Seed Development (Golden Seed Project no. 213007-05-4-SB510) of the Ministry of Agriculture, Food and Rural Affairs in the Republic of Korea (MAFRA).

## Conflict of Interest

The authors declare that the research was conducted in the absence of any commercial or financial relationships that could be construed as a potential conflict of interest.
