# Supplementary material for: Leptosphaeria maculans Alters Glucosinolate Accumulation and Expression of Aliphatic and Indolic Glucosinolate Biosynthesis Genes in Blackleg Disease-Resistant and -Susceptible Cabbage Lines at the Seedling Stage
Source: Front Plant Sci. 2020 Jul 30;11:1134. doi: 10.3389/fpls.2020.01134 (PMC7406797; doi:10.3389/fpls.2020.01134)
Supplement: Supplementary file 1 [file DataSheet_1.docx]

***Leptosphaeria maculans* alters glucosinolate accumulation and expression of aliphatic and indolic glucosinolate biosynthesis genes in blackleg disease–resistant and –susceptible cabbage lines at the seedling stage**

Arif Hasan Khan Robin^1, 2^, Rawnak Laila^1^, Md. Abuyusuf^1,3^, Jong-In Park^1^, and Ill-Sup Nou^1*^

Supplementary Materials

Table S1. Degrees of freedom, test statistic and P value of statistical significance for relative expression of *glucosinolate biosynthesis genes in* in four blackleg disease resistant and susceptible cabbage lines (two resistant and two susceptible lines) under 10 different treatments and time-points combinations at the seedling stage of cabbage plants.

| **Sl. No.** | **Variable** | **Treatment** | **df** | **Mean squares** | **F value** | **P value** |
| --- | --- | --- | --- | --- | --- | --- |
| 1 | *ST5b* (Bol026201) | Genotype | 3 | 43.7 | 385.4 | <0.01 |
|  |  | Treatment | 9 | 2.53 | 22.3 | <0.01 |
|  |  | Interaction | 27 | 2.59 | 22.9 | <0.01 |
| 2 | *ST5b* (Bol026202) | Genotype | 3 | 51.8 | 182.4 | <0.01 |
|  |  | Treatment | 9 | 151.4 | 533.5 | <0.01 |
|  |  | Interaction | 27 | 70.1 | 247.0 | <0.01 |
| 3 | *GSL-OH* (Bol033373) | Genotype | 3 | 27.5 | 52.7 | <0.01 |
|  |  | Treatment | 9 | 34.5 | 66.2 | <0.01 |
|  |  | Interaction | 27 | 13.3 | 25.5 | <0.01 |
| 4 | *MYB34* (Bol007760) | Genotype | 3 | 2178.4 | 33.7 | <0.01 |
|  |  | Treatment | 9 | 2816.2 | 43.6 | <0.01 |
|  |  | Interaction | 27 | 2600.9 | 40.3 | <0.01 |
| 5 | *ST5a* (Bol026200) | Genotype | 3 | 1511.1 | 69.1 | <0.01 |
|  |  | Treatment | 9 | 1781.1 | 81.4 | <0.01 |
|  |  | Interaction | 27 | 829.5 | 37.9 | <0.01 |
| 6 | *CYP81F4* (Bol032712) | Genotype | 3 | 574.7 | 108.6 | <0.01 |
|  |  | Treatment | 9 | 602.7 | 113.9 | <0.01 |
|  |  | Interaction | 27 | 426.1 | 80.3 | <0.01 |
| 7 | *CYP81F4* (Bol032714) | Genotype | 3 | 61.3 | 33.5 | <0.01 |
|  |  | Treatment | 9 | 82.8 | 45.3 | <0.01 |
|  |  | Interaction | 27 | 80.7 | 44.2 | <0.01 |
| 8 | *CYP81F2* (Bol026044) | Genotype | 3 | 3966.0 | 120.2 | <0.01 |
|  |  | Treatment | 9 | 3204.3 | 97.1 | <0.01 |
|  |  | Interaction | 27 | 1535.8 | 46.5 | <0.01 |

**Table S2.** Degrees of freedom, test statistic and P value of statistical significance glucosinolate contents in four blackleg disease resistant and susceptible cabbage lines (two resistant and two susceptible lines) under 7 different treatments and time-points combinations at the seedling stage of cabbage plants.

| **Sl. No.** | **Variable** | **Treatment** | **df** | **Mean squares** | **F value** | **P value** |
| --- | --- | --- | --- | --- | --- | --- |
| 1 | Glucoiberin | Genotype | 3 | 103.8 | 141.0 | <0.01 |
|  |  | Treatment | 6 | 14.4 | 19.6 | <0.01 |
|  |  | Interaction | 18 | 10.6 | 14.4 | <0.01 |
| 2 | Progoitrin | Genotype | 3 | 0.048 | 6.53 | <0.01 |
|  |  | Treatment | 6 | 0.015 | 2.06 | 0.07 |
|  |  | Interaction | 18 | 0.079 | 10.7 | <0.01 |
| 3 | Glucoraphanin | Genotype | 3 | 5.20 | 235.6 | <0.01 |
|  |  | Treatment | 6 | 0.2 | 9.24 | <0.01 |
|  |  | Interaction | 18 | 0.169 | 7.67 | <0.01 |
| 4 | Sinigrin | Genotype | 3 | 513.5 | 104.5 | <0.01 |
|  |  | Treatment | 6 | 107.8 | 21.9 | <0.01 |
|  |  | Interaction | 18 | 94.5 | 19.2 | <0.01 |
| 5 | Gluconapin | Genotype | 3 | 3.12 | 7.97 | <0.01 |
|  |  | Treatment | 6 | 2.17 | 5.54 | <0.01 |
|  |  | Interaction | 18 | 3.23 | 8.26 | <0.01 |
| 6 | Glucoiberverin | Genotype | 3 | 58.1 | 766.8 | <0.01 |
|  |  | Treatment | 6 | 5.53 | 72.9 | <0.01 |
|  |  | Interaction | 18 | 4.50 | 59.5 | <0.01 |
| 7 | Glucoerucin | Genotype | 3 | 51.9 | 89.6 | <0.01 |
|  |  | Treatment | 6 | 15.8 | 27.3 | <0.01 |
|  |  | Interaction | 18 | 25.8 | 44.6 | <0.01 |
| 8 | 4-hydroxyglucobrassicin | Genotype | 3 | 0.03 | 0.7 | 0.55 |
|  |  | Treatment | 6 | 0.25 | 5.59 | <0.01 |
|  |  | Interaction | 18 | 0.14 | 3.18 | <0.01 |
| 9 | Glucobrassicin | Genotype | 3 | 448.8 | 148.8 | <0.01 |
|  |  | Treatment | 6 | 112.2 | 37.2 | <0.01 |
|  |  | Interaction | 18 | 122.2 | 40.5 | <0.01 |
| 10 | 4-Methoxyglucobrassicin | Genotype | 3 | 53.8 | 126.9 | <0.01 |
|  |  | Treatment | 6 | 5.2 | 12.2 | <0.01 |
|  |  | Interaction | 18 | 22.2 | 52.3 | <0.01 |
| 11 | Neoglucobrassicin | Genotype | 3 | 1483.0 | 130.7 | <0.01 |
|  |  | Treatment | 6 | 187.7 | 16.5 | <0.01 |
|  |  | Interaction | 18 | 106.6 | 9.4 | <0.01 |
| 12 | Total glucosinolate | Genotype | 3 | 3943.4 | 79.2 | <0.01 |
|  |  | Treatment | 6 | 968.7 | 19.5 | <0.01 |
|  |  | Interaction | 18 | 989.2 | 19.9 | <0.01 |


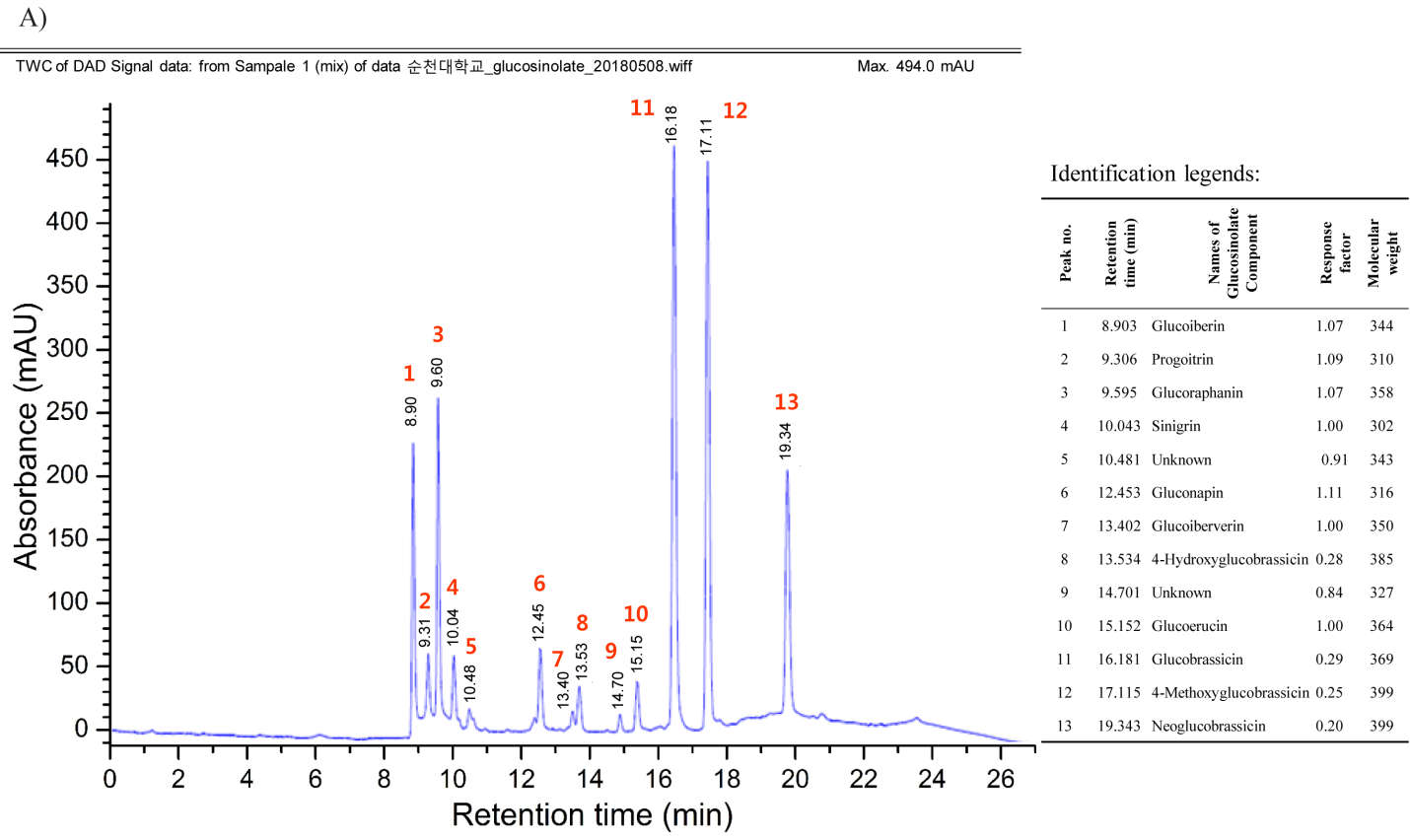


**Figure S1.** An illustrative spectrum to detect glucosinolate profiles of cabbage leaf samples from mass spectrometry analysis (HPLC/MS, Agilent 1200 series, Agilent Technologies). Retention time of each GSL compound along with corresponding response factor and molecular weight are presented in ‘identification legends’ (Abuyusuf et al., 2018a).
